# Ultra-Processed Food Intake and Risk of Insomnia: A Systematic Review and Meta-Analysis

**DOI:** 10.3390/nu16213767

**Published:** 2024-11-01

**Authors:** Ali Pourmotabbed, Farhang Hameed Awlqadr, Sanaz Mehrabani, Atefeh Babaei, Alexei Wong, Seyed Mojtaba Ghoreishy, Sepide Talebi, Mohammad Ali Hojjati Kermani, Faramarz Jalili, Sajjad Moradi, Reza Bagheri, Fred Dutheil

**Affiliations:** 1 Department of Physiology, School of Medicine, Kermanshah University of Medical Sciences, Kermanshah 6714869914, Iran; apourmotabbed@yahoo.com (A.P.); atefeh.babaie@gmail.com (A.B.); 2 Department of Food Science and Quality Control, Halabja Technical College, Sulaimani Polytechnic University, Sulaymaniyah 46001, Iraq; farhang.hamid.a@spu.edu.iq; 3 Nutrition and Food Security Research Center, Isfahan University of Medical Sciences, Isfahan 8174765191, Iran; sanimehrabani@gmail.com; 4 Department of Health and Human Performance, Marymount University, Arlington, VA 22207, USA; awong@marymount.edu; 5 Department of Nutrition, School of Public Health, Iran University of Medical Sciences, Tehran 1449614535, Iran; seyed.mojtaba.ghoreishy@gmail.com; 6 Student Research Committee, School of Public Health, Iran University of Medical Sciences, Tehran 1449614535, Iran; 7 Students’ Scientific Research Center (SSRC), Tehran University of Medical Sciences, Tehran 1439955991, Iran; talebisepide7@gmail.com; 8 Department of Clinical Nutrition, School of Nutritional Sciences and Dietetics, Tehran University of Medical Sciences, Tehran 1439955991, Iran; 9 Clinical Tuberculosis and Epidemiology Research Center, National Research Institute of Tuberculosis and Lung Diseases (NRITLD), Masih Daneshvari Hospital, Shahid Beheshti University of Medical Sciences, Tehran 1983969411, Iran; imhojjati@gmail.com; 10 School of Health Administration, Faculty of Health, Dalhousie University, Halifax, NS B3H 4R2, Canada; faramarz_jalili@yahoo.com; 11 Department of Nutrition and Food Sciences, Research Center for Evidence-Based Health Management, Maragheh University of Medical Sciences, Maragheh 7815155158, Iran; 12 Department of Exercise Physiology, University of Isfahan, Isfahan 8174673441, Iran; will.fivb@yahoo.com; 13 Université Clermont Auvergne, CNRS, LaPSCo, Physiological and Psychosocial Stress, CHU Clermont-Ferrand, University Hospital of Clermont-Ferrand, Preventive and Occupational Medicine, Witty Fit, F-63000 Clermont-Ferrand, France; fred_dutheil@yahoo.fr

**Keywords:** ultra-processed foods, insomnia, sleep, meta-analysis

## Abstract

Objectives: The objective of this investigation was to compile existing observational research and quantify the potential association between ultra-processed foods (UPFs) and the risk of insomnia using meta-analysis. Setting: We conducted a systematic search of the PubMed/MEDLINE, Scopus, and ISI Web of Science databases with no restrictions until 29 June 2024. Odds ratios (OR) and 95% confidence intervals (CI) were aggregated using a random-effects model, while the Newcastle-Ottawa Scale and Egger’s regression asymmetry test assessed study quality and publication bias, respectively. Results: Analysis of data from seven studies showed a significant positive association between higher intake of UPFs and an increased risk of insomnia (OR = 1.53; 95% CI: 1.20, 1.95; I^2^ = 62.3%; *p* = 0.014). Subgroup analysis indicated this positive relationship was particularly strong under the NOVA food classification (OR = 1.57; 95% CI: 1.03, 2.40; I^2^ = 78.5%; *p* = 0.009; n = 3) and with snack intake (OR = 1.33; 95% CI: 1.04, 1.71; I^2^ = 0.0%; *p* < 0.001; n = 2), compared to adherence to Western dietary patterns. Moreover, subgroup analysis based on age group showed that higher UPF intake was significantly associated with increased risk of insomnia among adolescents (OR = 1.55; 95% CI: 1.21, 1.99; I^2^ = 57.4%; *p* < 0.001) but not in adults. Conclusions: Our findings underscore a significant association between higher consumption of UPFs and increased risk of insomnia, particularly among adolescents. Further research is necessary to explore the intricacies of this association and to ensure the generalizability of these results.

## 1. Introduction

Insomnia is a common sleep disorder affecting diverse populations, with prevalence rates ranging from 5.8% to 32.8% [1,2]. It is influenced by demographic factors such as age, gender, marital status, income, and education [3]. Symptoms include difficulty falling asleep, waking during the night, and early morning awakenings, often leading to daytime distress or functional impairment [4,5]. This highlights the complex relationship between insomnia and overall health, emphasizing the need for a deeper understanding of its various causes and impacts on well-being.

The etiology of insomnia remains complex and not entirely elucidated, yet emerging research underscores its association with detrimental long-term consequences. These include heightened risks of psychological conditions like depression [6,7], workplace absenteeism [8,9], medical conditions such as hypertension [10], and even reduced lifespan [11]. Key risk factors for insomnia include increasing age, genetic predisposition, female gender, lifestyle habits, psychological stress and anxiety, socioeconomic status, and underlying medical and mental health conditions [12]. This recognition of insomnia’s widespread and multifaceted impact sets the stage for investigating other potential contributing factors, such as dietary habits. Diet has been implicated in both causing insomnia and being a significant factor in determining the quality of sleep [13,14,15,16]. The Mediterranean diet, which is distinguished by its high consumption of fruits, vegetables, whole grains, fish, and healthy fats, has been linked to a reduction in the symptoms of insomnia [17]. A study found that higher sugar consumption was associated with a greater incidence of insomnia among postmenopausal women [18]. Particularly, the intake of ultra-processed food (UPF), defined as heavily industrially processed items containing artificial additives and high levels of sugar, fat, or salt with low nutritional fiber, has been implicated in influencing both the onset and quality of sleep [19,20,21]. Indeed, a recent meta-analysis of cross-sectional studies conducted among children, adolescents, and adults found a statistically significant correlation between a high consumption of UPFs and sleep-related outcomes [22]. Moreover, Andreeva et al., in a systematic review showed that consuming UPFs was associated with negative effects on sleep parameters [23]. However, while several studies support a link between high UPF intake and an elevated possibility of experiencing insomnia [24,25,26], others have not conclusively demonstrated this relationship [27,28].

Despite extensive research, no prior meta-analysis has specifically explored the relationship between UPF consumption and insomnia risk. Our research endeavors to address the issue by compiling extant observational research and quantitatively evaluating the potential association between the consumption of UPFs and the risk of insomnia through meta-analysis.

## 2. Methods

The present study adhered to the Meta-analysis Of Observational Studies in Epidemiology (MOOSE) guidelines [29] and received approval from the PROSPERO registry (registration number CRD42024568758).

### 2.1. Literature Search and Selection

A systematic and exhaustive search was conducted across the ISI Web of Science, Scopus, and PubMed/MEDLINE databases (imposing no date restrictions) up to 29 June 2024. The search strategy utilized MeSH terms and procedures detailed in Supplementary Table S1. Grey literature was sourced through a manual search of references cited in primary studies indexed in the aforementioned databases.

### 2.2. Inclusion and Exclusion Criteria

Inclusion criteria were defined as follows: (a) observational studies (cohort, case-control, or cross-sectional) that reported data on the association between UPF intake and the risk of insomnia, providing effect estimates in the form of odds ratios (ORs), relative risk (RR), or hazard ratio (HR) with at least a 95% confidence interval (95% CI). Exclusion criteria were the following: (a) studies lacking relevant exposure and (b) studies without pertinent outcomes. Titles, abstracts, and subsequently, full text of potentially relevant studies were screened by two independent reviewers (SM and AB), with discrepancies resolved through consensus. The PICOS information is illustrated in Supplementary Table S2.

### 2.3. Data Extraction

Two researchers conducted data extraction independently, compiling (a) first author’s name, publication year, and country of origin; (b) study characteristics (design and data source); (c) participant characteristics (number, age, and sex); (d) insomnia assessment tools; (e) UPF evaluation methods; (f) primary findings (outcomes); and (g) covariates adjusted for multivariate analyses. Discrepancies were resolved by consensus.

### 2.4. Quality Assessment

Two researchers performed the quality assessment of each selected article employing the Newcastle-Ottawa Scale (NOS) [30], which evaluates non-randomized studies in systematic reviews and meta-analyses across three domains: selection (four points), comparability (two points), and outcome (three points). Studies were awarded a maximum of nine points, with 7–9 indicating high quality/low risk of bias and 0–3 suggesting high risk. The consensus on quality ratings is presented in Table 1.

### 2.5. Statistical Analyses and Data Synthesis

Statistical analyses were carried out employing STATA version 14.0 (StataCorp, College Station, TX, USA). The OR and 95% CI were established as the primary effect sizes in the current study, paralleling the effect estimates reported by the original investigations adhering to this meta-analysis’s inclusion criteria [34]. When original studies have reported several models of adjustment, we included the last adjusted model with further possible confounder control. The synthesized effect estimates for this investigation were reported as pooled OR with 95% CI. Due to anticipated heterogeneity between studies, effect estimates were calculated using the DerSimonian-Laird weighted random-effects model [35]. A pairwise meta-analysis was undertaken by pooling the effect size outcomes derived from the highest and lowest consumption categories of UPFs. Heterogeneity in the studies was assessed by Cochran’s Q and the I-squared (I^2^) statistics, where the I^2^ value was estimated from [(Q − df)/Q × 100%], Q being the χ^2^ value and df the corresponding degrees of freedom. Between-study heterogeneity was deemed significant if the Cochran’s Q statistic was significant (*p* < 0.01) or if I^2^ > 50%; heterogeneity was classified as low, moderate, high, and extreme based on I^2^ thresholds of <25%, 25–50%, 50–75%, and >75%, respectively.

Subgroup analyses were conducted to explore potential effects attributable to variables such as study design (cross-sectional, case-control, or cohort), UPF classification methodology (Western-diet pattern, NOVA food classification, snack food), racial/ethnic groups (Middle East, Latin), gender (male, female), age group (adults, adolescents), number of participants (<5000 or ≥5000), dietary assessment tools (Food Frequency Questionnaires [FFQ], brief diet history questionnaire), and other adjustments for covariates. Sensitivity analyses were performed by sequentially excluding each study and re-evaluating the remaining pooled effect estimates. Publication bias was evaluated through visual inspection of funnel plots and formally tested using Egger’s regression asymmetry test [36], with significance thresholds set at *p* < 0.05.

### 2.6. Quality of Evidence

The quality of evidence was assessed following the Grading of Recommendations Assessment, Development, and Evaluation (GRADE) guidelines. This approach evaluates the certainty of evidence by considering factors such as risk of bias, inconsistency, indirectness, imprecision, and publication bias. Based on these criteria, the quality of evidence is classified into four levels: high, moderate, low, and very low. This systematic framework ensures a transparent and structured assessment to support reliable conclusions and recommendations [37]. For the evaluation and rating of the evidence, S.T. and S.M. each used GRADE independently.

## 3. Results

### 3.1. Study Characteristics

**Figure 1:**
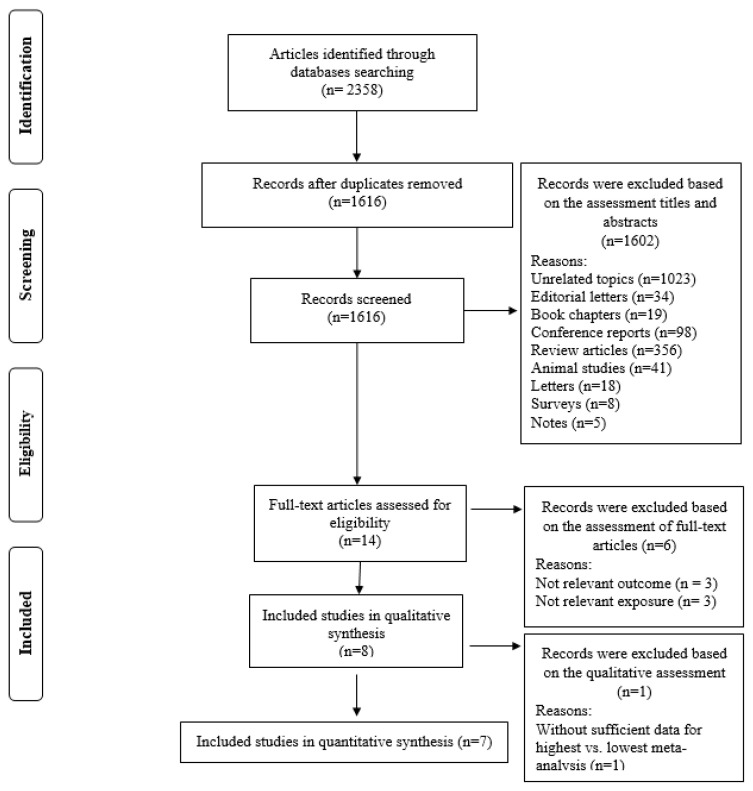
Flow chart of the process of the study selection.

The systematic search and reference list screening identified 2358 records. After omitting duplicates, 1616 studies remained for evaluation (Figure 1). Initial screening of titles and abstracts excluded 1602 records, leaving 14 for full-text assessment. Subsequent full-text reviews excluded six studies due to irrelevance to the research question (n = 3) or inadequate consideration of relevant exposure (n = 3) as detailed in Supplementary Table S3. In qualitative synthesis, one study excluded due to without sufficient data for highest vs. lowest meta-analysis [33]. Ultimately, seven studies met our inclusion criteria and were included in the quantitative analysis [24,25,26,27,28,31,32].

All the included studies (detailed in Table 1) had a cross-sectional design and were conducted between 2014 and 2024 across France [33], Iran [25,26,27,31,32], Brazil [24], and Mexico [28], comprising a cumulative sample of 159,427 participants. The studies variably focused on adult (n = 4) [26,28,32,33] and adolescent (n = 4) [24,27,31,38] populations. The Newcastle-Ottawa tool, applied for quality assessment, categorized three articles as high quality [24,31,33] and five as medium quality [25,26,27,28,32]. In addition, the results demonstrated that the level of agreement between researchers for data collection as well as for quality evaluation was appropriate (Kappa = 0.841).

### 3.2. Ultra-Processed Food Intake and Risk of Insomnia

**Figure 2:**
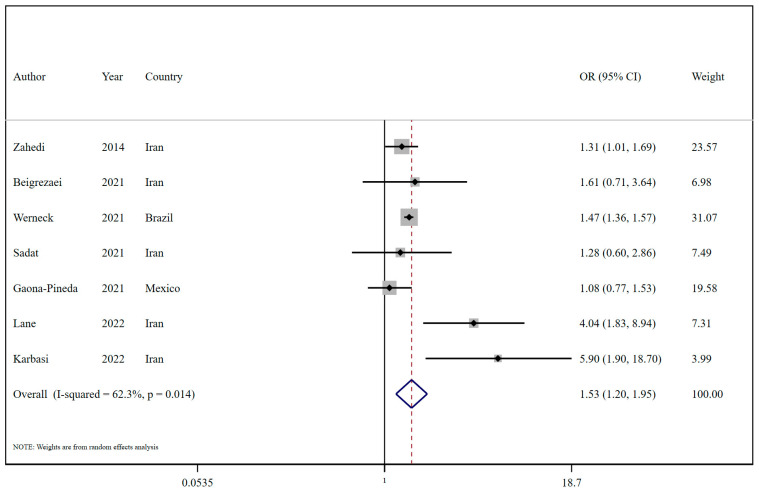
Forest plots demonstrating OR and 95% CI of pooled results from the random-effects models to evaluate the relationship between ultra-processed food consumption and risk of insomnia [24,25,26,27,28,31,32].

The outcomes indicated a significant positive relationship between higher UPF intake and an enhanced risk of insomnia (OR = 1.53; 95% CI: 1.20, 1.95; I^2^ = 62.3%; *p* = 0.014; n = 7; Figure 2) (Refer to Table 2 and Figure 1). Subgroup analysis also suggested that this positive relationship was observed in the context of NOVA food classification (OR = 1.57; 95% CI: 1.03, 2.40; I^2^ = 78.5%; *p* = 0.009; n = 3) and snack intake (OR = 1.33; 95% CI: 1.04, 1.71; I^2^ = 0.0%; *p* < 0.001; n = 2), in contrast to Western dietary pattern adherence. Moreover, in subgroup analysis by geographic origin, studies from the Middle East reported an OR of 2.05 (95% CI: 1.19 to 3.56; I^2^ = 68.6%; *p* = 0.013; n = 5), while Latin American studies found a non-significant association (OR = 1.32, 95% CI: 0.99 to 1.76; I^2^ = 66.3%; *p* = 0.085; n = 2). For sex, female participants showed a stronger association (OR = 3.18, 95% CI: 1.51 to 6.71; I^2^ = 51.3%; *p* = 0.128; n = 3), compared to studies including both sexes (OR = 1.39, 95% CI: 1.24 to 1.56; I^2^ = 17.1%; *p* = 0.305; n = 4) (Table 3). Subgroup analysis based on the number of participants revealed a statistically significant association between UPF intake and an increased risk of insomnia in studies with <5000 participants (OR = 2.49; 95% CI: 1.26, 4.94; I^2^ = 59.6%; *p* = 0.009; n = 4), but not in larger studies. Diet-assessment methods varied, with brief dietary history tools showing significant effects (OR = 1.46; 95% CI: 1.36, 1.56; I^2^ = 0.0%; *p* < 0.001; n = 3) in contrast to FFQs (Table 3).

### 3.3. Sensitivity Analyses and Publication Bias

**Figure 3:**
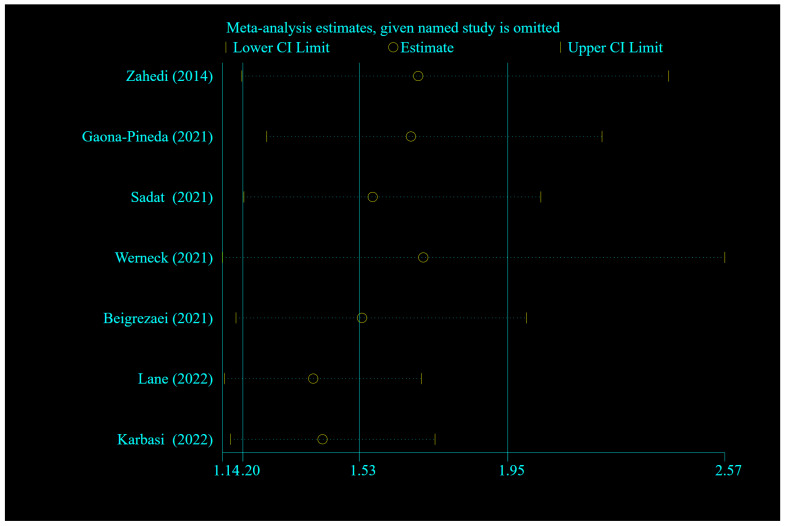
Forest plots showing sensitivity analysis results of the relationship between ultra-processed food intake and the risk of insomnia [24,25,26,27,28,31,32].

**Figure 4:**
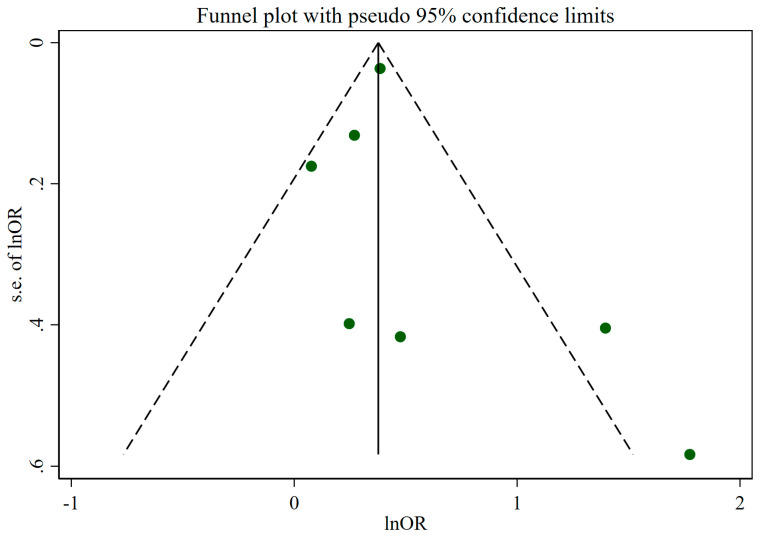
Funnel plot for evaluation of publication bias. Abbreviations: OR, Odds ratio.

Sensitivity analysis did not demonstrate a significant influence of any individual study on the overall meta-analysis results for insomnia (Figure 3). Egger’s test showed no evidence of publication bias (*p* = 0.498), although the funnel plot displayed some asymmetry, indicating potential variability in study effects regarding UPF intake and the risk of insomnia (Figure 4).

### 3.4. Quality of Evidence Results

The application of the GRADE tool rated the quality of evidence regarding the relationship between UPF intake and insomnia risk as low (Table 4). The GRADE system assesses the certainty of evidence based on several factors, including risk of bias, inconsistency, indirectness, and imprecision. The evidence was downgraded for serious risk of bias due to the cross-sectional design of included studies, which limits causal inference and raises the possibility of reverse causation. Additionally, many studies had insufficient control for confounding variables, such as lifestyle and dietary habits, further compromising the reliability of the findings. Serious inconsistency was observed with an I^2^ value of 62.3%, indicating substantial variability across studies. Although indirectness and imprecision were not considered serious, the large sample size (107,194 participants) provided a stable odds ratio of 1.53 (95% CI: 1.20 to 1.95). While the evidence suggests a significant association between UPF intake and insomnia, the low certainty rating highlights the need for further research with longitudinal or interventional designs to confirm these findings.

## 4. Discussion

The growing body of evidence continues to highlight the critical role of dietary patterns in and their implications for public health. Epidemiological research has identified a significant correlation between sleep disorders and adverse health outcomes. In exploring the link between dietary habits and insomnia, this investigation aimed to evaluate the impact of UPF consumption on the risk of developing insomnia, collecting data from multiple observational studies. The findings revealed a notable correlation between increased consumption of UPFs and an increased risk of insomnia. Furthermore, subgroup analyses demonstrated a significant relationship between UPF consumption and insomnia risk, particularly within the framework of the NOVA food classification system and snack intake, as contrasted with adherence to a Western dietary pattern. Additional subgroup findings indicated that elevated consumption of UPFs was significantly associated with a higher risk of insomnia in adolescents, whereas no significant correlation was found in adults.

Our study revealed a statistically significant positive linear association between increased UPF intake and enhanced risk of insomnia. This observation aligns with previous research, including the study by Lane et al. [25], which indicated a higher incidence of insomnia among individuals who are categorized as higher consumers of UPFs. Additionally, a cross-sectional analysis utilizing data from the NutriNet-Santé study demonstrated that UPF consumption was linked to an increased likelihood of chronic insomnia [33]. In line with these results, Werneck et al. [24] revealed a correlation between daily UPF consumption and an elevated probability of experiencing sleep disruptions triggered by anxiety. To expand on our research and the results of earlier observational studies, it is crucial to investigate the potential impact of ultra-processed foods (UPFs) on the disruption of normal physiological processes associated with sleep.

The potential link between the consumption of UPFs and the prevalence of insomnia might be explicated by several underlying biological mechanisms. Dietary melatonin plays a crucial role in regulating the sleep–wake cycle, and it is produced from dietary tryptophan through the synthesis of serotonin [39]. Empirical evidence suggests that ingestion of foods rich in tryptophan and melatonin can play a role in improving insomnia [40,41]. A study revealed that a lower dietary intake of tryptophan, specifically below the first quartile, was linked to a heightened risk of insomnia as measured by the Athens Insomnia Scale [42]. Additionally, dietary patterns characterized by high nutrient and fiber content (exemplified by the Mediterranean diet) have been associated with a reduced likelihood of developing insomnia [43]. There is also evidence of a causal association between the composition of the gut microbiota and insomnia [44]. It appears that the intake of UPFs could disrupt the balance of gut microbiota [45,46], leading to sleep disturbances like insomnia.

Moreover, the intake of sugar-sweetened beverages has been linked to reduced sleep duration [47]. The elevated dietary glycemic index and glycemic load, primarily driven by the increased consumption of added sugars and refined grains typical in UPFs, are hypothesized to contribute to insomnia development. The association between high glycemic index and glycemic load diets and increased risk of insomnia was revealed previously [48,49]. The mechanism posited involves the dietary glycemic index impacting blood glucose levels; high glycemic foods may cause rapid increases in blood glucose (leading to transient hyperglycemia), which could contribute to feelings of sleepiness and potentially disrupt normal sleep patterns. Postprandial hyperglycemia, resulting from a high dietary glycemic load, can lead to compensatory hyperinsulinemia, which may subsequently reduce plasma glucose levels [50]. This decline in glucose concentration can compromise brain function and trigger the release of autonomic counter-regulatory hormones [51]. The physiological response to these hormonal changes can manifest in various symptoms, such as anxiety, irritability, heart palpitations, tremor, and increased hunger [52]. Furthermore, a higher consumption of UPFs is associated with increased saturated fat intake [53], and prior research indicates that higher consumption of saturated fat and reduced intake of dietary fiber may reduce slow-wave sleep and increase nighttime arousals [17]. In addition, endocrine-disrupting chemicals (EDCs) such as bisphenol A (BPA), frequently found in the packaging of UPFs, have been implicated in sleep disturbances [54]. BPA exposure could potentially lead to the development of sleep disorders through its influence on cardiometabolic risk factors [55,56,57,58].

Our subgroup analysis demonstrated a significant association between the consumption of UPFs and the risk of insomnia, particularly when using the NOVA food classification system and focusing on snack intake rather than adherence to a Western dietary pattern. The NOVA classification categorizes UPFs based on the level and purpose of industrial food processing rather than nutritional content, providing the rationale for the observation that studies using this classification cover a broader spectrum of UPFs consumed by individuals [59]. Moreover, differential consumption patterns were noted across age demographics, with adolescents exhibiting a significant correlation between higher UPF intake and an elevated risk of insomnia. This association was not evident among adults. It appears that the adolescent diet has a higher proportion of processed foods compared to that of adults [60]. Previous research indicated that the elderly population exhibited the highest proportion of unprocessed or minimally processed foods in their dietary intake, with adults coming in a close second [60]. Further subgroup analyses highlighted a notable association between daily UPF consumption and a greater likelihood of insomnia compared to weekly consumption. Daily intake potentially results in increased exposure to harmful constituents such as additives, preservatives, excessive sugars, and unhealthy fats, which may have more negative health implications than sporadic (weekly) consumption [38]. Additionally, the application of brief diet history assessment tools in subgroup analysis yielded significant results, contrasting with findings from FFQs. It seems that brief dietary history tends to focus on specific dietary habits, potentially providing a more accurate evaluation of the correlation between UPF consumption and insomnia. For instance, the study by Zahedi et al. employed a brief questionnaire to delineate the frequency of participants’ consumption of junk foods, including sweets, sweetened beverages, fast foods, and salty snacks [31].

This systematic review and meta-analysis possess several strengths. A notable strength of this study is its comprehensive examination of the relationship between the consumption of ultra-processed foods (UPFs) and the risk of insomnia, achieved by analyzing all available observational data. This comprehensive methodology not only deepens our understanding of how UPF intake may influence insomnia risk but also strengthens the overall findings by integrating diverse research perspectives. However, it is important to acknowledge the inherent limitations of this research. Variabilities in the definitions and methods used to assess UPF intake across studies could affect the consistency and reliability of the findings. Moreover, the reliance on self-reported dietary data may lead to underestimations of UPF intake and be susceptible to recall bias. Additionally, the observational nature of the studies included limits the ability to establish causality; they can only suggest potential associations. Furthermore, the intake of UPFs and the development of insomnia can be influenced by a variety of factors, such as lifestyle choices, socioeconomic factors, and concurrent health conditions. In addition, this study, due to a lack of sufficient data, did not evaluate the dose–response association between UPFs and insomnia; hence, these relationships remain to be examined in next studies. Even with meticulous research methodologies, accounting for all potential sources of bias can be a complex and demanding endeavor.

## 5. Conclusions

The findings of this study indicate a significant link between increased UPF consumption and a higher risk of insomnia. Additionally, subgroup analyses exposed specific patterns of association: (I) A stronger association between UPF consumption and the risk of insomnia was noted in studies that utilized the NOVA food classification system and focused on snack consumption as opposed to those that examined adherence to a Western dietary pattern. (II) Daily consumption of UPFs was associated with a higher risk of insomnia compared to weekly intake. (III) Brief diet history assessment tools yielded significant outcomes, unlike the FFQ tool. (IV) A notably higher likelihood of insomnia was observed in adolescents compared to adults.

Our results underscore the importance of considering dietary factors in the prevention and treatment of insomnia. Public health initiatives should promote healthier eating practices, emphasizing the reduction of UPF intake to enhance sleep quality and overall health. Further research is necessary to elucidate the complexities of this association and to confirm the generalizability of the findings.

## Figures and Tables

**Table 1:** Characteristics of included studies.

Author (Year; Location)	Study Design/Follow up (Years)/Source of Data/Health Status	Population/ Age/(Women/Men)	Insomnia Assessment Method	Ultra-Processed Foods Assessment Method	Outcomes	Adjusted Variables	Quality Score
Zahedi et al. (2014, Iran) [31]	Cross-sectional study/CASPIAN-IV	N = 13,486/ Age = 12.47 ± 3.36 years/ (6640/6846)	Study questionnaire	Dietary behavior questionnaire/Sweets, sweetened beverages, fast foods, and salty snacks	Higher sweetened beverages, fast foods, and salty snack consumption were associated with risk of insomnia	Age, sex, BMI, family history of chronic diseases, mother’s education, screen time, physical activity, socioeconomic status	0.7
Sadat et al. (2020, Iran) [32]	Cross-sectional study	N = 444/ Age = 31.77 ± 9.99 years/ (349/95)	ISI	FFQ/Western dietary pattern	Higher adherence to Western dietary pattern was not associated with risk of insomnia	Age, sex, marital status, education, SES, BMI, smoking, physical activity GHQ score, energy intake	0.6
Beigrezaei et al. (2021, Iran) [27]	Cross-sectional study	N = 988/ Age = 14.52 ± 1.52 years/ (NR/NR)	ISI	Dietary behavior questionnaire/Consumption of fried foods and snacks	Fried food and snack intake was not associated with risk of insomnia	Age, menstruation, parent’s death, parent’s divorce, parent’s employment status, physical activity, BMI, energy intake	
Werneck et al. (2021, Brazil) [24]	Cross-sectional study/Adolescent School-Based Health Survey	N = 99,791/ Age = 14.3 years (range 11–19)/ (52,015/47,776)	Study questionnaire	Study questionnaire/The NOVA classification	Higher UPF consumption was associated with risk of insomnia	Age group, ethnicity, food insecurity, country region, type of city, physical activity	0.8
Gaona-Pineda et al. (2021, Mexico) [28]	Cross-sectional study/National Health and Nutrition Survey	N = 5076/ Age = 20–59/ (3340/1736)	Study questionnaire	FFQ/Industrialized dietary pattern	Higher adherence of Industrialized dietary pattern was not associated with risk of insomnia	Age, sex, body mass index, rural/urban area type, geographical region, physical activity level, lifetime tobacco use, tertiles of well-being index, total energy intake	0.6
Karbasi et al. (2022, Iran) [26]	Cross-sectional study	N = 159/ Age = 20.9 ± 1.7/ (159/0)	ISI	FFQ/Western dietary pattern	Higher adherence to Western dietary pattern was not associated with risk of insomnia	Age, BMI, WHR, depression, anxiety, stress, daytime sleepiness, cognitive abilities	0.5
Lane et al. (2022, Iran) [25]	Cross-sectional study	N = 733/ Age = 14.51 *±* 1.57/ (NR/NR)	ISI	FFQ/Australian processed food classification system	Higher UPF consumption was associated with risk of insomnia	Age, energy intake, BMI, physical activity	0.6
Duquenneet et al. (2024, France) [33]	Cross-sectional study/NutriNet-Santé study	N = 38,570/ Age = 50.0 ± 14.8 years/ (29,699/8871)	DSM-5 and ICSD-3		Higher UPF consumption was associated with risk of insomnia	Age, sex, socio-professional category, BMI, marital status, physical activity level, sedentariness, smoking status, alcohol consumption, energy intake, healthy and Western dietary patterns, diagnosis or treatment for anxiety and depression	0.8

Abbreviations. BMI, body mass index; FFQ, food-frequency questionnaire; GHQ, General Health Questionnaire; SES, socioeconomic status, DSM-5, Diagnostic and Statistical Manual of Mental Disorders; ICSD-3, International Classification of Sleep Disorders—3rd Edition; ISI, Insomnia Severity Index; UPF, ultra-processed food; WHR, waist to hip ratio.

**Table 2:** Dietary ultra-processed food and the risk of insomnia.

		OR (95% CI)	*p* Value	I^2^, %	*P* _heterogeneity_
Insomnia	7	1.53 (1.20, 1.95)	0.001	62.3	0.014

Abbreviations: OR; Odds ratio, CI; Confidence Interval.

**Table 3:** Subgroup analyses of ultra-processed food intake and the risk of insomnia (Highest vs. lowest category meta-analysis).

Sub-Groups	Number of Effect Sizes	Odds Ratio (95% CI), *p* Value	I^2^ (%), *P*_heterogeneity_	*P* _between_
Overall	7	1.53 (1.20, 1.95), 0.001	62.3, 0.014	
Ultra-processed food assessment method				<0.001
NOVA food classification	3	1.57 (1.03, 2.40), 0.035	78.5, 0.009	
Western-diet pattern	2	2.59 (0.58, 11.52), 0.212	76.8, 0.037	
Snack food	2	1.33 (1.04, 1.71), 0.021	0.0, 0.637	
Origin				0.021
Middle East	5	2.05 (1.19, 3.56), 0.010	68.6, 0.013	
Latin	2	1.32 (0.99, 1.76), 0.057	66.3, 0.085	
Sex				0.004
Female	3	3.18 (1.51, 6.71), 0.002	51.3, 0.128	
Both	4	1.39 (1.24, 1.56), <0.001	17.1, 0.305	
Age groups				0.303
Adults	3	1.73 (0.76, 3.93), 0.191	74.3, 0.020	
Adolescents	4	1.55 (1.21, 1.99), 0.001	57.5, 0.070	
Number of participants				0.026
<5000	4	2.49 (1.26, 4.94), 0.009	59.6, 0.059	
>5000	3	1.36 (1.16, 1.59), <0.001	43.3, 0.171	
Dietary assessment method				<0.001
FFQ	4	2.18 (0.98, 4.83), 0.056	80.2, 0.002	
Brief diet history	3	1.46 (1.36, 1.56), <0.001	0.0, 0.680	
Adjustments				
Energy intake				0.580
Yes	4	1.64 (0.92, 2.91), 0.092	67.2, 0.027	
No	3	1.52 (1.14, 2.04), 0.005	68.9, 0.040	
Smoking status				0.082
Yes	2	1.11 (0.81, 1.52), 0.515	0.0, 0.696	
No	5	1.76 (1.28, 2.41), <0.001	68.5, 0.013	
Sex				0.069
Yes	3	1.23 (1.00, 1.50), 0.045	0.0, 0.674	
No	4	2.42 (1.26, 4.63), 0.008	74.6, 0.008	

**Table 4:** GRADE evidence table for ultra-processed food intake and risk of insomnia.

Certainty Assessment					№ of Patients	Effect	Certainty	Importance
Risk of Bias	Inconsistency	Indirectness	Imprecision	Other Considerations	(n)	Odds Ratio (95% CI)		
serious ^a^	serious ^b^	not serious	not serious	none	107,194	1.53 (1.20 to 1.95)	⨁⨁◯◯ Low	IMPORTANT

Abbreviations: CI: Confidence interval. Explanations: ^a^. Downgraded since most studies judged as serious risk of bias based on NOS were included in the meta-analysis and residual confounding cannot be ruled out. ^b^. Serious inconsistency since I^2^ = 62.3%; downgraded. ⨁: Upgraded score; ◯: Downgraded score.

## Data Availability

The datasets are available from the corresponding author on reasonable request.

## Supplementary Materials

The following supporting information can be downloaded at https://www.mdpi.com/article/10.3390/nu16213767/s1, Table S1: Search strategies including the key terms and the queries for each database; Table S2: Description of population, intervention, comparator and outcome (PICOs); Table S3: Reason for exclusion of retrieved articles.
