# Supplementary material for: Ultra-Processed Food Intake and Risk of Insomnia: A Systematic Review and Meta-Analysis
Source: Nutrients. 2024 Nov 1;16(21):3767. doi: 10.3390/nu16213767 (PMC11548003; doi:10.3390/nu16213767)
Supplement: Supplementary file 1 [file nutrients-16-03767-s001.zip › Supplementary Table S1.pdf]

Table S1. Search strategies including the key terms and the queries for each database

Database<br>29 June 2024<br>6/29/2024	key terms and the queries
PubMed<br>(n=468)	#1 "Fast Foods"[MeSH Terms] OR "Candy"[MeSH Terms] OR "Ice Cream"[MeSH Terms] OR "Chocolate"[MeSH Terms] OR "Snacks"[MeSH Terms] OR "Feeding Behavior"[MeSH Terms] OR "ultra-processed"[Title/Abstract] OR "ultraprocessed"[Title/Abstract] OR "Fast Foods"[Title/Abstract] OR "processed food"[Title/Abstract] OR "ultraprocessed food"[Title/Abstract] OR "ultra-processed food"[Title/Abstract] OR "processed meat"[Title/Abstract] OR "ultra-processed food"[Title/Abstract] OR "ham"[Title/Abstract] OR "sausages"[Title/Abstract] OR "hamburger"[Title/Abstract] OR "bacon"[Title/Abstract] OR "luncheon meats"[Title/Abstract] OR "ready-to-eat"[Title/Abstract] OR "ready-to-consume"[Title/Abstract] OR "industrialized"[Title/Abstract] OR "fast-food"[Title/Abstract] OR "fast-food"[Title/Abstract] OR "fast-food"[Title/Abstract] OR "junk food"[Title/Abstract] OR "prepared food"[Title/Abstract] OR "Candy"[Title/Abstract] OR "Ice Cream"[Title/Abstract] OR "Chocolate"[Title/Abstract] OR "Snacks"[Title/Abstract] OR "hot dog"[Title/Abstract] OR "burger"[Title/Abstract] OR "dietary patterns"[Title/Abstract] OR "dietary behaviors"[Title/Abstract] OR "dietary habits"[Title/Abstract] OR "NOVA"[Title/Abstract] OR "nova food*"[Title/Abstract] OR "NOVA food classification system"[Title/Abstract]<br><br>#2 "Sleep Initiation and Maintenance Disorders"[Mesh] OR "Sleep Initiation and Maintenance Disorders"[tiab] OR "Sleeplessness"[tiab] OR "Insomnia Disorder"[tiab] OR "Insomnia Disorders"[tiab] OR "Insomnia"[tiab] OR "Insomnias"[tiab] OR "Chronic Insomnia"[tiab] OR "Psychophysiological Insomnia"[tiab]<br><br>#3 #1 AND #2
Web of Science (ISI)<br>(n=678)	# 1 TOPIC: ("ultra-processed" OR "ultraprocessed" OR "fast foods" OR "processed food" OR "ultraprocessed food" OR "ultra-processed food" OR "processed meat" OR "ultra processed food" OR "ham" OR "sausages" OR "hamburger" OR "bacon" OR "luncheon meats" OR "ready-to-eat" OR "ready-to-consume" OR "industrialized" OR "fast-food" OR "fast food" OR "fast food" OR "junk food" OR "prepared food" OR "candy" OR "ice cream" OR "chocolate" OR "snacks" OR "hot dog" OR "burger" OR "dietary patterns" OR "dietary behaviors" OR "dietary habits" OR "NOVA" OR "nova food classify*" OR "nova food*" OR "nova food classif*"OR "NOVA food classification system")<br><br>#2 TOPIC: ("Sleep Initiation and Maintenance Disorders") OR TOPIC: ("Sleep Initiation and Maintenance Disorders") OR TOPIC: (Sleeplessness) OR TOPIC: ("Insomnia Disorder") OR TOPIC: ("Insomnia Disorders") OR TOPIC: (Insomnia) OR TOPIC: (Insomnias) OR TOPIC: ("Chronic Insomnia") OR TOPIC: ("Psychophysiological Insomnia")<br>#3 #1 AND #2
Scopus<br>(n=1216)	#1 (TITLE-ABS-KEY ("Fast Foods") OR TITLE-ABS-KEY ("Candy") OR TITLE-ABS-KEY ("Ice Cream") OR TITLE-ABS-KEY ("Chocolate") OR TITLE-ABS-KEY (

	"Snacks") OR TITLE-ABS-KEY ("Feeding Behavior") OR TITLE-ABS-KEY ("ultra-processed") OR TITLE-ABS-KEY ("ultraprocessed") OR TITLE-ABS-KEY ("Fast Foods") OR TITLE-ABS-KEY ("processed food") OR TITLE-ABS-KEY ("ultraprocessed food") OR TITLE-ABS-KEY ("ultra-processed food") OR TITLE-ABS-KEY ("processed meat") OR TITLE-ABS-KEY ("ultra-processed food") OR TITLE-ABS-KEY ("ham") OR TITLE-ABS-KEY ("sausages") OR TITLE-ABS-KEY ("hamburger") OR TITLE-ABS-KEY ("bacon") OR TITLE-ABS-KEY ("luncheon meats") OR TITLE-ABS-KEY ("ready-to-eat") OR TITLE-ABS-KEY ("ready-to-consume") OR TITLE-ABS-KEY ("industrialized") OR TITLE-ABS-KEY ("fast-food") OR TITLE-ABS-KEY ("junk food") OR TITLE-ABS-KEY ("prepared food") OR TITLE-ABS-KEY ("Candy") OR TITLE-ABS-KEY ("Ice Cream") OR TITLE-ABS-KEY ("Chocolate") OR TITLE-ABS-KEY ("Snacks") OR TITLE-ABS-KEY ("hot dog") OR TITLE-ABS-KEY ("burger") OR TITLE-ABS-KEY ("dietary patterns") OR TITLE-ABS-KEY ("dietary behaviors") OR TITLE-ABS-KEY ("dietary habits") OR TITLE-ABS-KEY ("NOVA") OR TITLE-ABS-KEY ("nova food*") OR TITLE-ABS-KEY ("NOVA food classification system")<br><br>#2 (TITLE-ABS-KEY ("Sleep Initiation and Maintenance Disorders") OR TITLE-ABS-KEY ("Sleep Initiation and Maintenance Disorders") OR TITLE-ABS-KEY ("Sleeplessness") OR TITLE-ABS-KEY ("Insomnia Disorder") OR TITLE-ABS-KEY ("Insomnia Disorders") OR TITLE-ABS-KEY ("Insomnia") OR TITLE-ABS-KEY ("Insomnias") OR TITLE-ABS-KEY ("Chronic Insomnia") OR TITLE-ABS-KEY ("Psychophysiological Insomnia"))<br><br>#3 #1 AND #2
