# Supplementary material for: Ultra-Processed Food Intake and Risk of Insomnia: A Systematic Review and Meta-Analysis
Source: Nutrients. 2024 Nov 1;16(21):3767. doi: 10.3390/nu16213767 (PMC11548003; doi:10.3390/nu16213767)
Supplement: Supplementary file 1 [file nutrients-16-03767-s001.zip › Supplementary Table S2.pdf]

Table S2. Description of population, intervention, comparator and outcome (PICOs).

**Population**	All age groups
**Intervention**	None
**Comparison**	Ultra-processed Food Intake
**Outcome**	The risk of insomnia
**Study design**	Observational research (cross-sectional, case-control, or cohort)
