# Supplementary material for: Ultra-Processed Food Intake and Risk of Insomnia: A Systematic Review and Meta-Analysis
Source: Nutrients. 2024 Nov 1;16(21):3767. doi: 10.3390/nu16213767 (PMC11548003; doi:10.3390/nu16213767)
Supplement: Supplementary file 1 [file nutrients-16-03767-s001.zip › Supplementary Table S3.pdf]

Table S3. Reason for exclusion of retrieved articles

References	Reason for exclusion
1. Jiangli, L., et al., Association of sugar-sweetened beverage consumption with insomnia and depressive symptoms among first-grade middle school students from Yunnan Province. 中国学校卫生, 2024. 45(2): p. 173-177.	Not relevant outcome
2. Kleppang, A.L., et al., Physical activity, sugar-sweetened beverages, whole grain bread and insomnia among adolescents and psychological distress in adulthood: prospective data from the population-based HUNT study. International Journal of Behavioral Nutrition and Physical Activity, 2021. 18: p. 1-11	
3. Werneck, A.O., et al., *Joint association of ultra-processed food and sedentary behavior with anxiety-induced sleep disturbance among Brazilian adolescents.* Journal of Affective Disorders, 2020. **266**: p. 135-142.	
4. Wang, S., et al., The Obesity-Related Dietary Pattern Is Associated with Higher Risk of Sleep Disorders: A Cross-Sectional Study from NHANES. Nutrients, 2022. 14(19).	Not relevant exposure
5. Katagiri, R., et al., *Low Intake of Vegetables, High Intake of Confectionary, and Unhealthy Eating Habits are Associated with Poor Sleep Quality among Middle-aged Female Japanese Workers.* Journal of Occupational Health, 2014. **56**(5): p. 359-368.	
6. Agostini, A., et al., Associations between self-reported sleep measures and dietary behaviours in a large sample of Australian school students. Journal of Sleep Research, 2018. 27(5): p. e12682-Article No.: e12682.	
7. Duquenne P, Capperella J, Fezeu LK, Srour B, Benasi G, Hercberg S, Touvier M, Andreeva VA, St-Onge MP. The Association Between Ultra-Processed Food Consumption and Chronic Insomnia in the NutriNet-Santé Study. J Acad Nutr Diet. 2024 Sep;124(9):1109-1117.e2. doi: 10.1016/j.jand.2024.02.015. Epub 2024 Feb 28. PMID: 38423510.	Without sufficient data for highest vs. lowest meta-analysis
